# Risk of developing juvenile idiopathic arthritis after quadrivalent HPV vaccination: a retrospective cohort study using the TriNetX U.S. Network

**DOI:** 10.3389/fimmu.2025.1621939

**Published:** 2025-08-25

**Authors:** Wen-Yu Wu, Hsin-Hua Chen, Ming-Chin Tsai, Yung-Chieh Huang, Jun-Peng Chen, Lin-Shien Fu

**Affiliations:** ^1^ Department of Pediatrics, Taichung Veterans General Hospital, Taichung, Taiwan; ^2^ Division of Allergy, Immunology and Rheumatology, Department of Medicine, Taichung Veterans General Hospital, Taichung, Taiwan; ^3^ Division of Clinical Informatics, Center for Quality Management, Taichung Veterans General Hospital, Taichung, Taiwan; ^4^ Department of Post-Baccalaureate Medicine, College of Medicine, National Chung Hsing University, Taichung, Taiwan; ^5^ Program in Translational Medicine and Rong Hsing Research Center for Translational Medicine, National Chung Hsing University, Taichung, Taiwan; ^6^ Big Data Center, National Chung Hsing University, Taichung, Taiwan; ^7^ Department of Pediatrics, National Yang Ming Chiao Tung University, Taipei, Taiwan; ^8^ Department of Research, Taichung Veterans General Hospital, Taichung, Taiwan

**Keywords:** quadrivalent human papillomavirus vaccine (HPV4 vaccine), juvenile idiopathic arthritis, TriNetX, vaccine safety, cohort study

## Abstract

**Introduction:**

Human papillomavirus (HPV) infection has been implicated in autoimmune processes, yet concerns remain about the potential autoimmune risks of HPV vaccination. Juvenile idiopathic arthritis (JIA) is a chronic autoimmune condition that typically manifests in childhood. The relationship between HPV vaccination and the development of JIA remains uncertain.

**Methods:**

We conducted a retrospective cohort study using data from the TriNetX U.S. Collaborative Network. Females aged 9–13 years were included. Three analyses were performed: (1) comparing HPV4-vaccinated vs. unvaccinated matched cohorts; (2) a stricter comparison excluding subjects with positive ANA; (3) comparing single vs. multiple HPV4 doses. Propensity score matching and Cox proportional hazards models were used to calculate hazard ratios (HRs) and 95% confidence intervals (CIs).

**Results:**

In Analysis 1 (n=55,257 pairs) and Analysis 2 (n=53,827 pairs), the HPV4-vaccinated groups showed significantly reduced rates of JIA from 12 to 36 months post-vaccination (HR range: 0.33–0.52). No difference in JIA risk was observed between single and multiple doses in Analysis 3 (n=20,822 pairs). Early-onset JIA (<6 months after HPV4 vaccine) showed inconsistent trends, with only limited protective signals.

**Conclusions:**

Our findings suggest that HPV4 vaccination is not associated with an increased risk of JIA. On the contrary, vaccination may confer a long-term protective effect against new-onset JIA, observable from 6–12 months and lasting for at least 3 years. These findings support the safety and possible immunoregulatory benefit of HPV4 in adolescents.

## 1 Introduction

Human papillomaviruses (HPVs) are double-stranded DNA viruses associated with a wide spectrum of human diseases, including cervical cancer and autoimmune conditions (1, 2). Prophylactic HPV vaccination, particularly the quadrivalent HPV4 vaccine, has demonstrated robust efficacy in preventing HPV-related infections and malignancies (3, 4). Despite its favorable safety profile, concerns remain regarding its potential role in triggering autoimmune disorders such as juvenile idiopathic arthritis (JIA).

JIA is the most prevalent autoimmune rheumatic disease in children, characterized by persistent joint inflammation without a known etiology. It is believed to result from complex interactions between genetic susceptibility and environmental triggers, including viral infections (5). Molecular mimicry and immune dysregulation have been proposed as key mechanisms by which pathogens may initiate or exacerbate autoimmunity (6, 7).

Previous studies have yielded conflicting findings on the association between HPV4 vaccination and the development of autoimmune arthritis. A U.S.-based analysis of the Vaccine Adverse Event Reporting System (VAERS) reported an elevated odds ratio for rheumatoid arthritis following HPV4 vaccination (8). Similarly, a Colombian study employing inverse probability of treatment weighting also observed increased arthritis risk (9). However, both relied on self-reported adverse events, potentially introducing reporting bias. Conversely, a retrospective study using electronic medical records from Kaiser Permanente found no significant increase in JIA incidence within 180 days post-vaccination (10).

To address these inconsistencies, we conducted a large-scale retrospective cohort study using the TriNetX U.S. Collaborative Network. This federated platform offers access to de-identified electronic health records across multiple healthcare organizations, enabling robust longitudinal analyses (11). We aimed to evaluate whether HPV4 vaccination is associated with an increased or decreased risk of new-onset JIA in adolescent females. Three complementary analyses were performed to examine the effect of vaccination status, antinuclear antibody (ANA) positivity, and HPV4 dose number on subsequent JIA risk.

## 2 Materials and methods

### 2.1 Data sources

Data were obtained from the TriNetX U.S. Collaborative Network, comprising 57 healthcare organizations with integrated electronic health records (EHRs). The study population included females aged 9 to 13 years between January 1, 2010 and December 31, 2015 (for Analyses 1 and 2) or up to December 31, 2017 (for Analysis 3). Three analyses were conducted as follows:

- Analysis 1: Compared HPV4-vaccinated individuals (n = 55,257) to an unvaccinated control cohort (n = 1,323,995). Individuals with a prior diagnosis of JIA (ICD-10: M08.xx), or prescription of methotrexate or antirheumatic drugs (**Supplementary Tables 1**, **2**), were excluded.
- Analysis 2: Built on the criteria in Analysis 1, with additional exclusion of individuals with positive ANA serology (ICD-10: R76.0 or R68.89). This analysis involved 53,827 vaccinated individuals and 1,399,261 unvaccinated controls.
- Analysis 3: Focused on HPV4-vaccinated individuals only, comparing those who received a single dose (n = 10,411) versus multiple doses (n = 10,411). The extended study period accounted for the 6–12 month dosing interval.

TriNetX’s integrated propensity score matching algorithm was used to match cohorts 1:1 based on age at index event (first vaccination or clinical visit), race (White, Black, Asian), ICD-10 diagnoses related to the musculoskeletal system (M00–M99), and prior NSAID use. Only individuals identified as White, Black or African American, or Asian were included in race-based matching, due to data completeness and sample size considerations.

### 2.2 Study design

**Figure 1:**
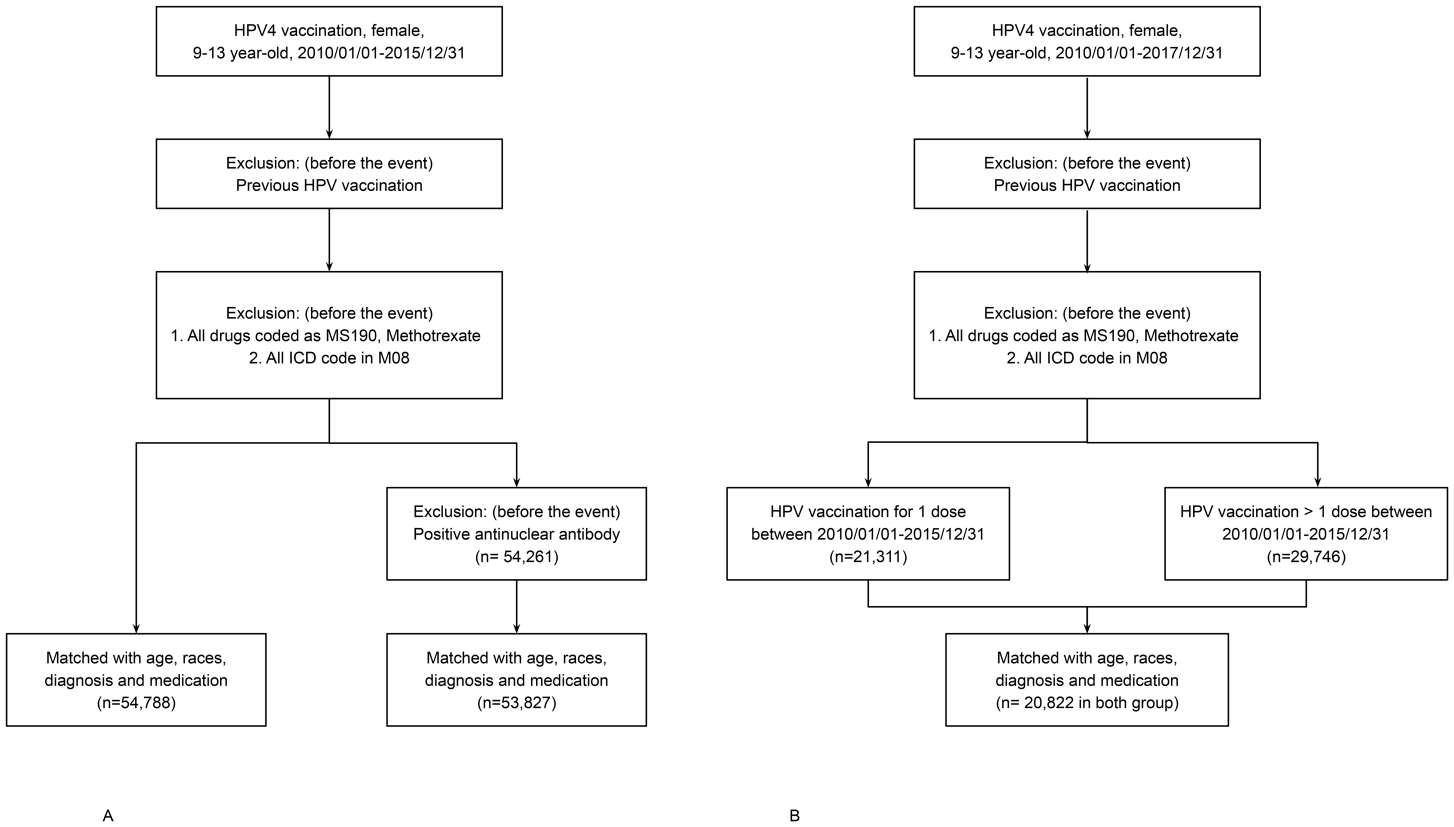
**(A)** Flowchart of cohort construction for Analyses 1 and 2. HPV4-vaccinated and unvaccinated individuals (n = 55,257 per group) were selected from the TriNetX U.S. Collaborative Network and matched 1:1 using propensity scores based on age at index event, race, prior musculoskeletal diagnoses, and NSAID use. Follow-up lasted up to 36 months post-index. **(B)** Flowchart of cohort construction for Analysis 3. HPV4-vaccinated individuals were stratified into single-dose and multiple-dose groups (n = 10,411 per group) using 1:1 propensity score matching with identical covariates. Follow-up continued for up to 36 months post-index.

As shown in **Figures 1A, B**, matched cohorts were followed for up to 36 months post-index event. Incident JIA was defined by ICD-10 code M08.xx, with or without concurrent use of disease-modifying antirheumatic drugs (DMARDs) or NSAIDs. Cases diagnosed within 7 days post-index event were excluded to avoid capturing pre-existing or unrelated conditions.

The number of new-onset JIA cases was assessed at intervals of 42 days, 3, 6, 12, 18, 24, 30, and 36 months post-event. Medication records were reviewed to confirm JIA diagnoses based on prescription of NSAIDs, methotrexate, or other DMARDs.

### 2.3 Ethical approval

The study protocol was approved by the Institutional Review Board of Taichung Veterans General Hospital (Approval No. CE23480C). Given the use of de-identified data, informed consent was waived. All data accessed through the TriNetX platform were de-identified in compliance with the Health Insurance Portability and Accountability Act (HIPAA) Privacy Rule, and no patient-level identifiers were available to the investigators.

### 2.4 Statistical analysis

To control for potential confounding, propensity score matching (1:1) was applied to balance baseline covariates between cohorts, including age at index event, race, prior musculoskeletal or connective tissue diagnoses (ICD-10 M00–M99), and NSAID use. Time-to-event analysis was performed using the Kaplan–Meier method based on daily intervals. Censoring was applied at the last recorded encounter. Hazard ratios (HRs) with 95% confidence intervals (CIs), log-rank tests, and proportionality assessments were generated through the TriNetX survival analysis module.

Due to the platform’s design, individual-level Schoenfeld residuals were unavailable; therefore, the proportional hazards assumption was indirectly assessed using cumulative incidence curves derived from daily JIA-free probabilities over a 36-month follow-up period. To account for multiple comparisons across time points, p-values were adjusted using the False Discovery Rate (FDR) method proposed by Benjamini and Hochberg. All statistical analyses were conducted using the TriNetX platform and R software (version 3.5; survival package). A two-tailed p-value < 0.05 was considered statistically significant.

## 3 Results

### 3.1 Cohort characteristics

The demographic and baseline clinical characteristics of the matched cohorts for all three analyses are presented in **Table 1**. In Analysis 1, 55,257 participants were included in both the HPV4-vaccinated and unvaccinated cohorts, with a mean age of 11.7 ± 1.0 years. The racial distribution was 51.0–51.1% White, 24.9% Black or African American, and 2.7% Asian. Baseline prevalence of musculoskeletal/connective tissue disorders was 18.9%, and NSAID use was noted in 15.8% of participants.

**Table 1:** Baseline characteristics of study subjects after matching.

Variables	Analysis 1				Analysis 2				Analysis 3			
	Vaccination group (n= 55,257)	Non-vaccination (n= 55,257)	p-value	SMD	Vaccination group (n= 53,827)	Non-vaccination (n= 53,827)	p-value	SMD	Vaccination 1/time (n= 20,822)	Vaccination>1/time (n= 20,822)	p-value	SMD
	n (%) or mean ± SD	n (%) or mean ± SD			n (%) or mean ± SD	n (%) or mean ± SD			n (%) or mean ± SD	n (%) or mean ± SD		
Age at index (y/o)	11.7 ± 1.0	11.7 ± 1.0	1	<0.001	11.7 ± 1.0	11.7 ± 1.0	1	<0.001	11.7 ± 1.0	11.7 ± 1.0	1	<0.001
Race or ethnicity												
White	27,917 (51.0)	28,005 (51.1)	0.6	0.003	27,392 (50.9)	27,470 (51.0)	0.6	0.003	10,254 (49.2)	10,245 (49.2)	0.93	0.001
Black or African American	13,625 (24.9)	13,625 (24.9)		<0.001	13,417 (24.9)	13,417 (24.9)	1	<0.001	5,485 (26.3)	5,437 (26.1)	0.59	0.005
Asian	1,468 (2.7)	1,468 (2.7)	1	<0.001	1,437 (2.7)	1,437 (2.7)	1	<0.001	495 (2.4)	498 (2.4)	0.92	0.001
Diagnosis												
Disease of the musculoskeletal system and connective tissue	10,349 (18.9)	10,349 (18.9)	1	<0.001	9,927 (18.4)	9,927 (18.4)	1	<0.001	3,472 (16.7)	3,460 (16.6)	0.88	0.002
Medication												
NSAIs	8,679 (15.8)	8,679 (15.8)	1	<0.001	8,295 (15.4)	8,295 (15.4)	1	<0.001	2,518 (12.1)	2,555 (12.3)	0.58	0.005

In Analysis 2, the matched vaccinated and unvaccinated groups each consisted of 53,827 individuals with similar age and race distributions. ANA-positive individuals were excluded. In Analysis 3, 20,822 HPV4-vaccinated individuals were divided into single-dose and multiple-dose cohorts (10,411 each). Demographics and clinical variables were well balanced following propensity score matching.

### 3.2 Incidence of new-onset JIA

The cumulative incidence and odds ratios (ORs) of new-onset JIA over 36 months are summarized in **Tables 2A–C**. JIA was defined either solely by ICD-10 M08.xx diagnosis (criterion 1) or by M08.xx with concurrent DMARD or NSAID usage (criterion 2). To account for multiple comparisons across time points, the False Discovery Rate (FDR) method adjusted p-values were also shown in **Table 2**.

**Table 2:** Incidence and odds ratio of new onset JIA and JIA with drug.

A. Analysis 1										
Analysis 1	JIA					JIA with Drug				
	Vaccination (%)	Non-vaccination (%)	OR (95% CI)	p-value	FDR p-value	Vaccination (%)	Non-vaccination (%)	OR (95% CI)	p-value	FDR p-value
8 days to 42 days	10 (0.018)	14 (0.026)	0.71 (0.32-1.60)	0.4153	0.4153	10 (0.018)	0 (0.000)	20.99(1.23-358.19)	0.0355	0.0473
8 days to 3 months	10 (0.018)	22 (0.040)	0.45 (0.22-0.96)	0.0385	0.0440	9 (0.016)	4 (0.007)	2.25 (0.69-7.30)	0.1776	0.2030
8 days to 6 months	10 (0.018)	30 (0.055)	0.33 (0.16-0.68)	0.0026	0.0035	9 (0.016)	12 (0.022)	0.75 (0.32-1.78)	0.5130	0.5130
8 days to 12 months	10 (0.018)	42 (0.077)	0.24 (0.12-0.47)	<0.0001	0.0003	6 (0.011)	22 (0.040)	0.27 (0.11-0.67)	0.0048	0.0122
8 days to 18 months	19 (0.035)	52 (0.095)	0.37 (0.22-0.62)	0.0002	0.0004	13 (0.024)	32 (0.058)	0.41 (0.21-0.77)	0.0061	0.0122
8 days to 24 months	26 (0.047)	61 (0.111)	0.43 (0.27-0.67)	0.0003	0.0005	19 (0.035)	37 (0.068)	0.51 (0.30-0.89)	0.0180	0.0288
8 days to 30 months	32 (0.058)	79 (0.144)	0.40 (0.27-0.61)	<0.0001	0.0003	26 (0.047)	54 (0.099)	0.48 (0.30-0.77)	0.0022	0.0122
8 days to 3 years	37 (0.068)	83 (0.152)	0.45 (0.30-0.66)	<0.0001	0.0003	31 (0.057)	59 (0.108)	0.52 (0.34-0.81)	0.0037	0.0122

JIA, Juvenile idiopathic arthritis.

JIA with Drug: JIA plus at least one drug use. The drugs included non-salicylate drugs, methotrexate and other anti-rheumatic drugs shown in Supplementary data.

FDR, False Discovery Rate (FDR) method proposed by Benjamini and Hochberg.

**Table:** 

B. Analysis 2										
Analysis 2	JIA					JIA with Drug				
	Vaccination (%)	Non-vaccination (%)	OR (95% CI)	p-value	FDR p-value	Vaccination (%)	Non-vaccination (%)	OR (95% CI)	p-value	FDR p-value
8 days to 42 days	10 (0.019)	12 (0.022)	0.83 (0.36-1.93)	0.6691	0.6691	10 (0.019)	0 (0.007)	2.45 (0.78-7.97)	0.1217	0.1623
8 days to 3 months	10 (0.019)	21 (0.039)	0.48 (0.22-1.01)	0.0532	0.0608	9 (0.017)	7 (0.013)	1.28 (0.48-3.45)	0.6189	0.7073
8 days to 6 months	10 (0.019)	30 (0.056)	0.33 (0.16-0.68)	0.0026	0.0035	9 (0.017)	9 (0.017)	0.99 (0.40-2.52)	0.9989	0.9989
8 days to 12 months	10 (0.019)	47 (0.087)	0.21 (0.11-0.42)	<0.0001	0.0002	6 (0.011)	22 (0.041)	0.27 (0.11-0.67)	0.0048	0.0096
8 days to 18 months	19 (0.035)	59 (0.110)	0.33 (0.20-0.55)	<0.0001	0.0002	13 (0.024)	32 (0.059)	0.41 (0.21-0.77)	0.0061	0.0098
8 days to 24 months	26 (0.048)	72 (0.134)	0.36 (0.23-0.57)	<0.0001	0.0002	19 (0.035)	43 (0.080)	0.44 (0.26-0.76)	0.003	0.0096
8 days to 30 months	32 (0.059)	83 (0.154)	0.39 (0.26-0.58)	<0.0001	0.0002	26 (0.048)	52 (0.097)	0.50 (0.32-0.80)	0.0039	0.0096
8 days to 3 years	36 (0.067)	91 (0.169)	0.40 (0.27-0.28)	<0.0001	0.0002	30 (0.056)	61 (0.113)	0.49 (0.32-0.76)	0.0014	0.0096

JIA, Juvenile idiopathic arthritis.

JIA with Drug: JIA plus at least one drug use. The drugs included non-salicylate drugs, methotrexate and other anti-rheumatic drugs shown in Supplementary data.

FDR, False Discovery Rate (FDR) method proposed by Benjamini and Hochberg.

**Table:** 

C. Analysis 3										
Analysis 3	JIA					JIA with Drug				
	Vaccination 1 dose (%)	Vaccination 2 or 3 doses (%)	OR (95% CI)	p-value	FDR p-value	Vaccination 1 dose (%)	Vaccination 2 or 3 doses (%)	OR (95% CI)	p-value	FDR p-value
8 days to 42 days	0 (0.000)	10 (0.048)	0.05 (0.003-0.81)	0.0354	0.2832	0 (0.000)	10 (0.048)	0.05 (0.003-0.81)	0.0354	0.2832
8 days to 3 months	10 (0.048)	10 (0.048)	1.00 (0.42-2.40)	0.9997	0.9997	9 (0.043)	10 (0.048)	0.90 (0.37-2.21)	0.8183	0.9352
8 days to 6 months	10 (0.048)	10 (0.048)	1.00 (0.42-2.40)	0.9997	0.9997	9 (0.043)	10 (0.048)	0.90 (0.37-2.21)	0.8183	0.9352
8 days to 12 months	10 (0.048)	10 (0.048)	1.00 (0.42-2.40)	0.9997	0.9997	9 (0.043)	7 (0.034)	1.29 (0.48-3.45)	0.6181	0.9352
8 days to 18 months	10 (0.048)	10 (0.048)	1.00 (0.42-2.40)	0.9997	0.9997	7 (0.034)	7 (0.034)	1.00 (0.35-2.85)	0.9998	0.9998
8 days to 24 months	10 (0.048)	13 (0.062)	0.77 (0.34-1.75)	0.5325	0.9997	7 (0.034)	8 (0.038)	0.87 (0.32-2.41)	0.7962	0.9352
8 days to 30 months	10 (0.048)	15 (0.072)	0.67 (0.30-1.48)	0.3203	0.8541	8 (0.038)	10 (0.048)	0.80 (0.32-2.03)	0.6378	0.9352
8 days to 3 years	11 (0.053)	18 (0.086)	0.61 (0.29-1.29)	0.1979	0.7916	8 (0.038)	13 (0.062)	0.62 (0.24-1.48)	0.2797	0.9352

JIA, Juvenile idiopathic arthritis.

JIA with Drug: JIA plus at least one drug use. The drugs included non-salicylate drugs, methotrexate and other anti-rheumatic drugs shown in Supplementary data.

FDR, False Discovery Rate (FDR) method proposed by Benjamini and Hochberg.

In Analysis 1, there was no significant difference in JIA incidence at 42 days post-vaccination (criterion 1: OR = 0.71, 95% CI = 0.32–1.60; criterion 2: OR = 20.99, 95% CI = 1.23–358.19). However, significantly lower JIA rates were observed in the HPV4 cohort at 3 and 6 months by criterion 1 (OR = 0.45 and 0.33, respectively), but not by criterion 2. From 12 to 36 months, both criteria consistently showed a significantly reduced JIA incidence in the vaccinated cohort (p < 0.05 for all time points).

**Figure 2:**
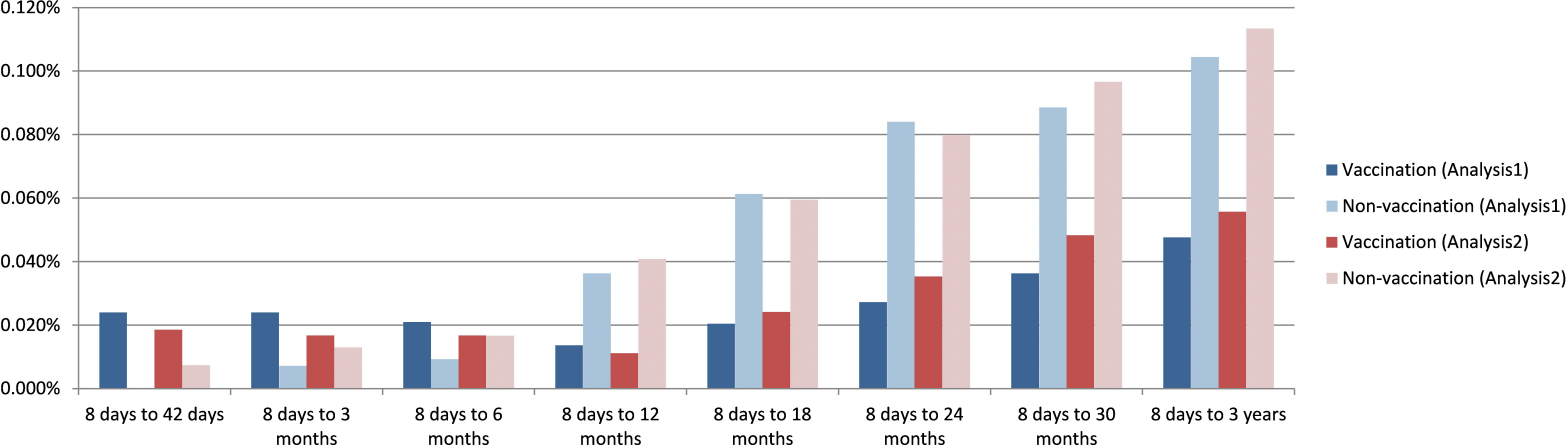
Rate of new onset juvenile arthritis defined by M08.xx plus drug use at 42 days, 3, 6, 9, 12, 24, 30, 36 months after the event (visit or HPV4 vaccination) in Analysis 1 and Analysis 2.

Analysis 2 showed a similar trend. No significant differences were observed at 42 days and 3 months, but a significant reduction in JIA incidence appeared from 6 months onward (criterion 1: OR = 0.33 at 6 months; p < 0.01). This protective effect remained consistent through 36 months (**Tables 2B**, **Figure 2**).

In Analysis 3, comparing single versus multiple HPV4 doses, no significant differences in JIA incidence were detected across all time points (**Table 2C**).

### 3.3 Survival and risk analyses

**Figure 3:**
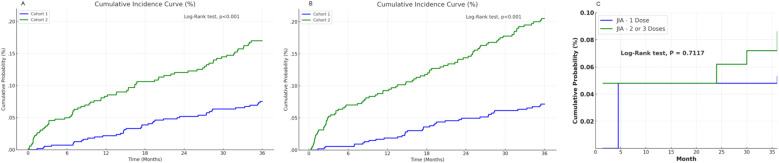
Cumulative probability curves for new onset juvenile idiopathic arthritis. **(A)** the curve derived from Analysis 1, **(B)** the curve derived from Analysis 2. **(C)** the curve derived from Analysis 3.

The cumulative incidence approach based on daily JIA-free probabilities allowed for time-resolved comparisons without assuming a constant hazard ratio over the entire follow-up period. The Cumulative probability curves comparing Cohorts 1 and 2 defined by criteria 1 for Analysis 1 and 2 are presented in **Figures 3A, B** (log-rank p < 0.0001). In Analysis 3, there was no difference in cumulative JIA incidence between single- and multiple-dose recipients (**Figure 3**, log-rank p = 0.717).

**Figure 4:**
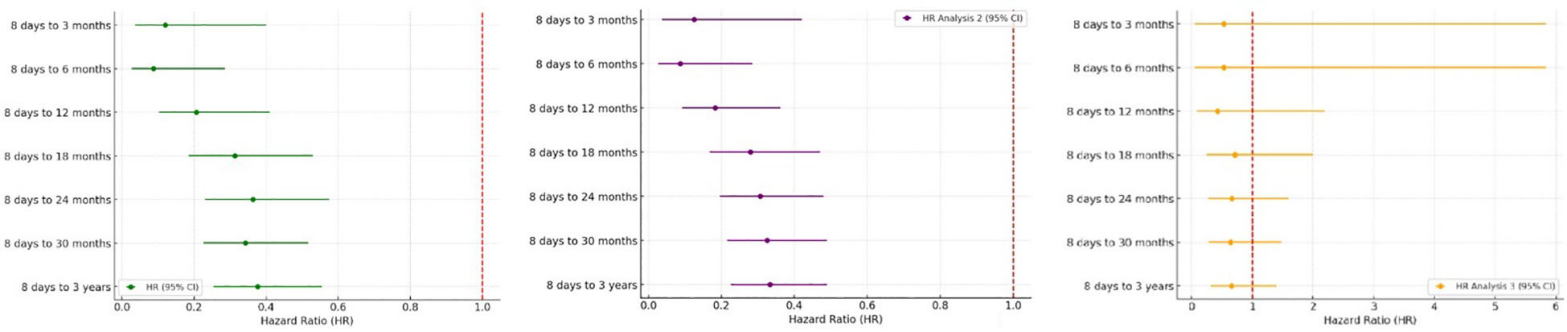
Forest Plots showed hazard ratios of new onset JIA in A. Analysis 1, B. Analysis 2 and C. Analysis 3.

Hazard ratios for JIA development are shown in **Figure 4** and **Table 3**. HPV4 vaccination was associated with reduced hazard across all post-vaccination intervals in Analyses 1 and 2. No significant difference in HRs was found in Analysis 3.

**Table 3:** Hazard ratio of new onset JIA in 3 analyses.

Time	Analysis 1		Analysis 2		Analysis 3	
	HR (95% CI)	p-value	HR (95% CI)	p-value	HR (95% CI)	p-value
8 days to 3 months	0.120 (0.036-0.400)	0.066	0.125 (0.037-0.420)	0.421	0.529 (0.048-5.833)	0.701
8 days to 6 months	0.087 (0.027-0.286)	0.970	0.087 (0.027-0.285)	0.718	0.529 (0.048-5.833)	0.905
8 days to 12 months	0.206 (0.103-0.410)	0.017	0.183 (0.092-0.362)	0.137	0.425 (0.082-2.191)	0.287
8 days to 18 months	0.313 (0.185-0.530)	0.001	0.280 (0.167-0.470)	0.001	0.713 (0.254-2.002)	0.902
8 days to 24 months	0.363 (0.230-0.575)	0.012	0.306 (0.196-0.480)	0.057	0.660 (0.273-1.592)	0.602
8 days to 30 months	0.343 (0.227-0.517)	0.386	0.325 (0.216-0.489)	0.384	0.644 (0.282-1.472)	0.759
8 days to 3 years	0.376 (0.255-0.554)	0.095	0.333 (0.226-0.489)	0.124	0.658 (0.311-1.394)	0.795

## 4 Discussion

This large-scale, multicenter retrospective cohort study found no evidence of increased risk of juvenile idiopathic arthritis (JIA) following quadrivalent HPV vaccination. In contrast, our findings consistently demonstrated a protective association between HPV4 vaccination and new-onset JIA, particularly from 12 months to 36 months post-vaccination. These results held true across two distinct analyses, including one that excluded ANA-positive individuals, thus strengthening the validity of the observed protective trend.

The absence of early-onset autoimmune flares (i.e., within 42 days) aligns with current immunological understanding, which suggests that an adaptive autoimmune response generally takes longer to manifest (12). The 42-day threshold is also the standard time frame used by the VAERS system for defining vaccine-associated arthritis (13). Therefore, our observation that JIA onset was significantly lower in the vaccinated cohort beginning at 6 to 12 months post-vaccination is biologically plausible and statistically robust.

Our findings corroborate prior research suggesting a potential autoimmune link between HPV infection and rheumatic diseases, including RA and systemic lupus erythematosus (14, 15). One tertiary center study showed that 48.2% of HPV DNA+ patients had autoantibody or autoimmune disease (16). The mechanism for their condition was generally considered to be via molecular mimicry and cross reactions between self-antigens and viral proteins (17). HPV-47, in particular, contains sequences with molecular mimicry to citrullinated autoantigens involved in RA pathogenesis (18). Moreover, HPV infection has been associated with altered immune cell activation, including Th17 cell expansion and elevated IL-17 levels, which may potentiate local and systemic inflammation (19).

One possible explanation for our findings is that HPV4 vaccination may reduce the systemic immune burden associated with persistent HPV infection, thereby lowering the probability of autoimmune activation in genetically predisposed individuals. This hypothesis is supported by recent NHANES data showing that HPV4 vaccination was associated with reduced RA incidence among adults aged 18–59 years (20).

Although the HPV4 vaccine is not classified as a tolerogenic vaccine, its nature as a non-live virus-like particle (VLP) formulation typically administered in multiple doses may favor regulatory T cell (Treg) induction under low-inflammatory conditions. Previous studies have suggested that repeated antigen exposure from inactivated or subunit vaccines can promote tolerogenic responses by providing persistent but non-replicative stimulation, which facilitates Treg differentiation via IL-10 production, reduced co-stimulation, and antigen presentation in tolerogenic dendritic cell contexts (21). However, in our study, no significant difference in JIA incidence was observed between individuals who received only one dose versus those who completed the full vaccination schedule. This may reflect the relatively small sample size in the single-dose subgroup, limiting the power to detect potential dose-dependent effects. Nonetheless, the possibility that even a single dose of HPV4 vaccine provides sufficient antigen exposure to initiate protective immune modulation cannot be excluded.

Our results are consistent with the known epidemiology of JIA in the United States. Previously reported incidence rates in girls aged 11–15 years range from 13.2 to 30.2 per 100,000 (22). In our study, the observed JIA incidence—based on both diagnostic coding and medication use—fell within or slightly above this range, affirming the external validity of our cohort. Importantly, using a more stringent diagnostic criterion (M08.xx plus drug use) revealed a delayed but more robust protective effect beginning at 6–12 months, likely reflecting improved specificity for true JIA cases. Taken together, the consistency between our observations and prior evidence—including NHANES epidemiologic data, molecular mimicry mechanisms, and the Treg-promoting potential of inactivated vaccines—provides additional support for the biological plausibility of a protective association.

This study has several limitations. First, the TriNetX dataset is predominantly U.S.-based and may not generalize to other populations, particularly Asian cohorts where sample sizes were limited. Second, temporal variation in vaccine administration may confound short-term effects, although this is unlikely to influence long-term outcomes. Third, while the use of both diagnostic codes and medication prescriptions enhances case validation, misclassification remains possible. Not all individuals with an M08.xx code received antirheumatic therapy, suggesting possible overcoding or transient arthropathy. As with all EHR-based studies, the potential for diagnostic misclassification exists; however, our strict case definitions and exclusion of pre-index autoimmune diagnoses or treatments help reduce such risk. Additionally, although extensive matching was performed, residual confounding from unmeasured variables—such as family history, HLA genotype, environmental exposures, and socioeconomic status —cannot be excluded. Although vaccination histories other than HPV were not available for analysis, routine immunizations are limited between ages 9 and 13, with seasonal influenza vaccine being the only widely administered immunization during this period.

In conclusion, this large-scale TriNetX study showed the protective effect which at least one dose of HPV4 vaccination from 12 months to 36 months has on developing JIA. Single or multiple doses of the vaccine showed similar rates of new onset JIA from 42 days to 3 years after HPV vaccination.

## Data Availability

The datasets presented in this study can be found in online repositories. The names of the repository/repositories and accession number(s) can be found in the article/**Supplementary Material**.

## Supplementary material

Supplementary Table 1 Drugs in MS109.

Supplementary Table 2 Diagnosis of ICD 10=M.08.xx.
