# Supplementary material for: Risk of developing juvenile idiopathic arthritis after quadrivalent HPV vaccination: a retrospective cohort study using the TriNetX U.S. Network
Source: Front Immunol. 2025 Aug 25;16:1621939. doi: 10.3389/fimmu.2025.1621939 (PMC12415063; doi:10.3389/fimmu.2025.1621939)
Supplement: Supplementary Table 1 — Drugs in MS109. [file Table1.docx]

Supplementary Table 1: Drugs in MS109.

| Drugs in MS109 |
| --- |
| Abatacept, Adalimumab, Anakinra, Apremilast, Baricitinib, Etanercept, Golimumab, Hyaluronate, Leflunomide, Risankizumab, Sarilumab, Tocilizumab, Upadacitinib |
