# Supplementary material for: Risk of developing juvenile idiopathic arthritis after quadrivalent HPV vaccination: a retrospective cohort study using the TriNetX U.S. Network
Source: Front Immunol. 2025 Aug 25;16:1621939. doi: 10.3389/fimmu.2025.1621939 (PMC12415063; doi:10.3389/fimmu.2025.1621939)
Supplement: Supplementary Table 2 — Diagnosis of ICD 10=M.08.xx. [file Table2.docx]

Supplementary Table 2: Diagnosis of ICD 10=M.08.xx.

| **Diagnosis of ICD 10=M.08.xx** | |
| --- | --- |
| M08 | Juvenile arthritis |
| M08.0 | Unspecified juvenile rheumatoid arthritis |
| M08.00 | Unspecified juvenile rheumatoid arthritis of unspecified site |
| M08.06 | Unspecified juvenile rheumatoid arthritis, knee |
| M08.09 | Unspecified juvenile rheumatoid arthritis, multiple sites |
| M08.2 | Juvenile rheumatoid arthritis with systemic onset |
| M08.20 | Juvenile rheumatoid arthritis with systemic onset, unspecified site |
| M08.3 | Juvenile rheumatoid polyarthritis (seronegative) |
| M08.4 | Pauciarticular juvenile rheumatoid arthritis |
| M08.40 | Pauciarticular juvenile rheumatoid arthritis, unspecified site |
| M08.461 | Pauciarticular juvenile rheumatoid arthritis, right knee |
| M08.8 | Other juvenile arthritis |
| M08.80 | Other juvenile arthritis, unspecified site |
| M08.86 | Other juvenile arthritis, knee |
| M08.89 | Other juvenile arthritis, multiple sites |
| M08.9 | Juvenile arthritis, unspecified |
| M08.90 | Juvenile arthritis, unspecified, unspecified site |
| M08.96 | Juvenile arthritis, unspecified, knee |
| M08.99 | Juvenile arthritis, unspecified, multiple sites |
